# Prevention of Remifentanil Induced Postoperative Hyperalgesia by Dexmedetomidine via Regulating the Trafficking and Function of Spinal NMDA Receptors as well as PKC and CaMKII Level In Vivo and In Vitro

**DOI:** 10.1371/journal.pone.0171348

**Published:** 2017-02-09

**Authors:** Yuan Yuan, Zhe Sun, Yi Chen, Yuxin Zheng, Ke-liang Xie, Ying He, Zhifen Wang, Guo-lin Wang, Yong-hao Yu

**Affiliations:** 1 Department of Anesthesiology, Tianjin Medical University General Hospital, Tianjin, China; 2 Tianjin Research Institute of Anesthesiology, Tianjin, China; Centre de Recherche Jean-Pierre Aubert, FRANCE

## Abstract

Remifentanil-induced secondary hyperalgesia has been demonstrated in both animal experiments and clinical trials. Enhancement of N-methyl-D-aspartate (NMDA) receptor trafficking as well as protein kinase C (PKC) and calmodulin-dependent protein kinase II (CaMKII) have been reported to be involved in the induction and maintenance of central sensitization. In the current study, it was demonstrated that dexmedetomidine could prevent remifentanil-induced hyperalgesia (RIH) via regulating spinal NMDAR-PKC-Ca^2+^/ CaMKII pathway in vivo and in vitro. We firstly investigated the effect of dexmedetomidine, a highly selective α2-adrenergic receptor agonist, on mechanical and thermal hyperalgesia using a rat model of RIH. NMDA receptor subunits (NR1, NR2A and NR2B) expression and membrane trafficking as well as PKC and CaMKII expression in spinal cord L4–L5 segments were measured by Western blot analysis. The expression of NMDA receptor subunits (NR1, NR2A and NR2B) were also detected by immunohistochemistry. Further more, the effect of dexmedetomidine on NMDA receptor current amplitude and frequency in spinal cord slices were investigated by whole-cell patch-clamp recording. We found that remifentail infusion at 1.2 μg.kg^−1^.min^−1^ for 90 min caused mechanical and thermal hyperalgesia, up-regulated NMDA receptor subunits NR1 and NR2B expression in both membrane fraction and total lysate as well as increased PKC and CaMKII expression in spinal cord dorsal horn. Subcutaneously injection of dexmedetomidine at the dose of 50 μg/kg at 30 min before plantar incision significantly attenuated remifentanil-induced mechanical and thermal hyperalgesia from 2 h to 48 h after infusion, and this was associated with reversal of up-regulated NR1 and NR2B subunits in both membrane fraction and total lysate as well as increased PKC and CaMKII expression in spinal cord dorsal horn. Furthermore, remifentanil incubation increased amplitude and frequency of NMDA receptor-induced current in dorsal horn neurons, which was dose-dependently attenuated by dexmedetomidine. These results suggest that dexmedetomidine can significantly ameliorate RIH via modulating the expression, membrane trafficking and function of NMDA receptors as well as PKC and CaMKII level in spinal dorsal horn, which present useful insights into the mechanistic action of dexmedetomidine as a potential anti-hyperalgesic agents for treating RIH.

## Introduction

As potent analgesics, opioids are available for relieving moderate to severe pain.

Meanwhile, their prolonged usage may paradoxically be related to the development of opioid-induced hyperalgesia (OIH) [1–3]. OIH is characterized by decreased pain threshold and increased sensitivity to noxious stimuli [2, 3]. Remifentanil is a μ-opioid receptor agonist for clinical anesthesia, however, it was claimed that remifentanil infusion might lead to OIH more frequently than other opioids because of having a rapid onset and short half-time of action [4].

Although the mechanisms underlying OIH have not been clearly illuminated, a certain amount of experiments suggest that it is associated with N-methyl-D-aspartate receptor (NMDAR)-protein kinase C(PKC)-Ca^2+^/calmodulin-dependent protein kinase II (CaMKII) pathway [5–8]. NMDAR, an ionotropic glutamate receptors, is a protein complex composed of three classes of subunits: the essential subunit NR1, the modulating subunit NR2 (A-D), NR3 (A, B). Membrane trafficking of NR1 and NR2B subunit has been observed in incisional pain rat model after remifentanil infusion through glycogen synthase kinase-3β (GSK-3β) pathway [9, 10]. PKC is a family of serine/threonine kinases distributed within the CNS, which consists of at least 12 isoforms [5]. Calcium dependent PKCγ isoform identified in lamina II of superficial spinal dorsal horn, an area implicated in pain, is considered to be a critical regulator of central sensitization by alleviating Mg^2+^ block of NMDA currents [6]. Ca^2+^/ CaMKII is a multifunctional serine/threonine protein kinase co-localized with the μ-opioid receptor in superficial spinal dorsal horn [7, 8]. It is implicated that CaMKIIα is a critical cellular signaling mechanism leading to and maintaining OIH [8].

Dexmedetomidine, a highly selective α2-adrenergic receptor (α2AR) agonist, possesses sedative, anxiolytic, analgesic, and hemodynamic-stabilizing properties [11, 12] without significant respiratory depression [13]. Its greater affinity to α2AR and shorter duration of action have led to its usage as an adjuvant for patients undergoing mechanical ventilation in general anesthesia [2,14]. It has been demonstrated that systemic administration of dexmedetomidine dramatically enhances analgesic effect of opioids and reduces perioperative analgesic requirements [11, 12, 15]. It has been suggested that antihyperalgesic effect of dexmedetomidine is linked to depression in spinal NMDAR-mediated synaptic transmission and dexmedetomidine may be an option to improve pain control for OIH patients [16, 17]. However, whether NMDAR-PKC-CaMKII pathway served as a target for preventing OIH was still needed to discuss.

The present study was conducted to investigate the efficacy of dexmedetomidine on remifentanil-induced postoperative hyperalgesia in an incisional pain rat model. We also wonder whether the antihyperalgesic effects of dexmedetomidine is associated with membrane trafficking of NMDAR and expression of PKC and CaMKII. In order to verify the effects, remifentanil was administrated intravenously and dexmedetomidine was administrated subcutaneously. The whole cell patch clamp was also applied to prove that whether miniature excitatory postsynaptic current (mEPSC) is relieved by dexmedetomidine via NMDA receptor pathway after management with remifentanil.

## Materials and methods

### Ethics statements

All experimental procedures and protocols were approved by the Institutional Animal Care Committee of Tianjin Medical University and performed according to the “Policies on the Use of Animal and Humans in Neuroscience Research”. The protocol was approved by the Committee on the Ethics of Animal Experiments of Tianjin Medical University General Hospital, Tianjin, China (Permit Number: 2015-X6-17). All surgery was performed under sevoflurane anesthesia, and all efforts were made to minimize suffering and to use the minimum number of animals necessary to obtain valid results.

### Animals

Both adult (weighing 240–260 g) and newborn (14–21 days old) male Sprague-Dawley rats, obtained from the Laboratory Animal Center of the Military Medical Sciences Academy of the Chinese People’s Liberation Army, were used in the experiment and housed with a 12 h light/12 h dark cycle. The rats were given free access to water and food ad libitum and kept in cages at a room temperature (18~22°C) and humidity (40%~60%) in a clean animal house at least 3 days before drug treatment or surgery. The experiment protocol was approved by the Institutional Animal Care and Use Committee of Tianjin Medical University and was performed on the basis of the National Institutes of Health Guide for Care and Use of Laboratory Animals. No efforts were spared to minimize animal sufferings.

### Experimental protocol

The rats were anesthetized under sevoflurane anesthesia (induction, 3.0%; maintenance, 1.0%; batch number: 100628; Maruishi Pharmaceutical Co., Osaka, Japan) via a nose mask. Then the rats were placed in plastic tubular restrainers. The middle and lower third of the tail was chosen as the puncture point. A 24-gauge venous indwelling needle filled with heparinized saline was inserted into the caudal vein.

1. To investigate whether dexmedetomidine could prevent remifentanil-induced hyperalgesia via regulating spinal NMDA receptor expression and membrane trafficking as well as PKC and CaMKII expression in vivo, 48 adult rats were randomly divided into 6 groups (n = 8 in each group): Blank control group (group C), Remifentanil plus incision group (group R+I, 1.2 μg.kg^−1^.min^−1^, 90 min, iv), Dexmedetomidine plus remifentanil and incision group(group D+R+I, dexmedetomidine: 50 μg/kg, 30 min before plantar incision, ih, remifentanil:1.2 μg.kg^−1^.min^−1^, 90 min, iv), Dexmedetomidine group(group D, dexmedetomidine: 50 μg/kg, ih), Remifentanil group (group R, 1.2 μg.kg^−1^.min^−1^, 90 min, iv), Incision group (group I, plantar incision). The thermal and mechanical hyperalgesia was evaluated by PWT and PWL at baseline (-24 h) and 2 h, 6 h, 24 h, 48 h after infusion. After the last behavioral test (48 h after infusion), spinal cord L4-L5 segments were harvested to evaluate NMDA receptor subunits (NR1, NR2A and NR2B) expression and membrane trafficking as well as PKC and CaMKII expression by western blot. The expression of NMDA receptor subunits (NR1, NR2A and NR2B) were also detected by immunohistochemistry.
2. To investigate whether dexmedetomidine could prevent remifentanil-induced hyperalgesia via regulating spinal NMDA receptor function in vitro, another 48 newborn SD rats were divided into 6 groups (n = 8 in each group) to run whole cell patch clamp recording test: Control group [group C, the spinal slices were only incubated with artificial cerebral spinal fluid (ACSF) for 90 min], Remifentanil group [group R, the spinal slices were incubated with artificial cerebral spinal fluid (ACSF) with 4 nM remifentanil for 90 min], Dexmedetomidine group [group D, the spinal slices were incubated with artificial cerebral spinal fluid (ACSF) with 4 nM dexmedetomidine for 90 min], Dexmedetomidine group 1 [group D1, the spinal slices were incubated with artificial cerebral spinal fluid (ACSF) with 4 nM remifentanil and 2 nM dexmedetomidine for 90 min], Dexmedetomidine group 2 [group D2, the spinal slices were incubated with artificial cerebral spinal fluid (ACSF) with 4 nM remifentanil and 4 nM dexmedetomidine for 90 min], Dexmedetomidine group 3 [group D3, the spinal slices were incubated with artificial cerebral spinal fluid (ACSF) with 4 nM remifentanil and 6 nM dexmedetomidine for 90 min]. After incubation, the NMDA receptor-mediated miniature excitatory postsynaptic current (mEPSC) was detected to evaluate the function of NMDA receptor.

### Plantar incision

The incisional pain rat model was constructed as described previously [18]. A 1-cm longitudinal incision was made through the skin, fascia, and muscle in sequence of the left hindpaw after disinfection, starting 0.5 cm from the proximal edge under sevoflurane anesthesia (induction, 3.0%; maintenance, 1.0%) via a nose mask. Pay attention to divide the underlying flexor muscle but keep the muscles origins and terminations and attachment integrity. The skin was sutured without inverted, overlap, cracked by 4–0 silk sutures after compression hemostasis. Finally, erythromycin ointment was smeared to avoid infection. Postoperative monitoring of temperature, heart rate and condition of the condition (bleeding, pyosis and so on) should be done every 2 h. If the incision of rat was suppurative or bleeding all the time, some analgesics should be given and the rat was excluded.

### Behavioral testing

Paw withdrawal threshold (PWT) was recorded by electronic von Frey filaments (BSEVF3, Harvard Apparatus, Edenbridge, UK) to measure mechanical hyperalgesia.

After adult rats were placed individually in mental cages (20 cm×20 cm×15 cm) with a wire grid bottom for 10 min, Von Frey filaments were exerted vertically to the plantar side of the right hindpaw. Lifting of the hindpaw off the cage surface or flinching was regarded as a positive response. A critical value of 60 g was used to prevent paw damage, and each trial was repeated 3 times at 10-min intervals. The calculated average value was PWT.

Paw withdrawal latency (PWL) was recorded by a 55°C hotplate (YLS-6B, Zheng Hua Biological Instrument Co., Anhui, China) to assess thermal hyperalgesia. A critical value of 60 seconds was used to prevent paw damage, and each trial was repeated 3 times at 10-min intervals. The calculated average value was PWL.

### Western blot

After completing the last behavioral tests, rats were sacrificed under sevoflurane anesthesia. The chest was cut along the midline to expose the heart, then an infusion needle was inserted into the apical portion of the aorta, the right atrial appendage was cut and normal saline was infused rapidly until transparent liquid flowed out. Then a longitudinal incision along the dorsal midline was made and L4-L5 spinal cord segments were extracted rapidly and kept in liquid nitrogen. The segments were homogenized in ice-cold sodium dodecyl sulfate lysis buffer containing protease inhibitors (Sigma-Aldrich Co., St. Louis, MO, USA). Then the lysate was centrifuged at 14,000r for 10 min at 4°C, and the supernatant was removed as total protein. A membrane compartment protein extraction kit (Biochain Institute, Inc., Hayward, CA, USA) was applied to extract the membrane protein. Eventually after the total protein was boiled at 95°C for 10min and membrane protein was boiled at 65°C for 10min, they were store at -20°C. Samples were separated on 8%-10% SDS-PAGE gel electrophoresis, and then transferred onto nitrocellulose membranes (Millipore, Billerica, MA). The membranes were blocked with 5% skim milk in TBST for 2 hour and subsequently incubated overnight at 4°C with mouse anti-rat epidermal growth factor receptor (EGFR, 1:1,000; MBL, Naka-ku Nagoya, Japan), monoclonal mouse anti-β-actin antibody (1:3000; Liangsen Biological Technology Co.,Ltd, Shanghai USA), polyclonal rabbit antibodies against rat NR1(1:500, Abcam, Cambridge, UK), NR2A, NR2B(1:1000, Abcam, Cambridge, UK), PKCγ(1:2000, Abcam, Cambridge, UK), CaMKIIα(1:5000, Abcam, Cambridge, UK), and polyclonal mouse antibodies against rat pCaMKIIα (1:2000, Abcam, Cambridge, UK) respectively. And then the membranes were incubated with horseradish peroxidase-conjugated goat anti-rabbit and goat anti-mouse IgG antibodies (1:5000, KPL, A SeraCare Company, USA) for 1 h. The density of each band was detected by a chemiluminescence imaging system and measured by Gene Tools Match software (Syngene, Cambridge, UK). The percentage of endogenous control (EGFR or β-actin) immunoreactivity was expressed as the results.

### Immunohistochemistry

The L4-L5 spinal cord segments were extracted rapidly after the last behavioral test. 4% paraformaldehyde in phosphate buffered saline (PBS) was applied to fix the samples for 6 h, and then paraffin was used to embed the samples. The samples were cut into 7-μm-thick sections and placed onto glass slides. After deparaffinization and rehydration in descending concentrations of ethanol, the sections were treated with avidin-biotin peroxidase for 15 min. To make the results more intuitive, citrate solution was used for antigen retrieval. 5% normal goat serum was selected for treatment to block non-specific reactions for 1h at room temperature. Then the sections were incubated with primary antibody of rabbit polyclonal anti-NR1, NR2A, NR2B (1:200; Abcam, Cambridge, UK) overnight at 4°C respectively. After rewarming for 45min, the sections were incubated with biotinylated secondary antibody (1:300, Boster Biological Technology, Ltd., Wuhan, China) for 30 min at room temperature and stained by diaminobenzidine (DAB substrate kit, Boster Biological Technology). Sections were counterstained with hematoxylin, differentiated by ethanol hydrochloride, dehydrated in ascending series of ethanol, cleared in xylene and coverslipped with neutral gum. Images were obtained from an Olympus eclipse 80i microscope (Olympus, Tokyo, Japan).

### Spinal cord slice preparation and whole-cell patch-clamp recording

Rat spinal cord slices were prepared as described previously. The postnatal (14–21 days old) rats were anesthetized with sevoflurane (induction, 3.0%; maintenance, 1.0%). The L4–L5 spinal cord segments were separated rapidly with the same method preciously, sliced into transverse slices (400 μm) with a vibratome (VT1000S, Leica, Germany), then incubated in ACSF at room temperature (22°C-25°C) and aerated with 95% O_2_ and 5% CO_2_ at pH 7.4 for 1 h. ASCF is composed of (in mM): 126 NaCl, 3.5 KCl, 2 MgCl_2_, 2 CaCl_2_, 1.25 NaH_2_PO_4_, 26 NaHCO_3_ and 10 D-glucose. The slices were then transferred to a recording chamber in succession. The chamber was perfused with oxygenated ACSF continuously and placed on an upright microscope (BX51W1, Olympus, Tokyo, Japan) and continuously perfused with oxygenated ACSF. Individual neurons were identified by a television monitor connected to the low light-sensitive CCD camera (710M, DVC, USA). Borosilicate glass microelectrodes with tip openings of 1–2 μm and electric resistance of 3–5 MΩ were drawn by vertical electrode puller (PIP5, HEKA, Germany) and applied to whole-cell patch-clamp recording. Microelectrodes were filled with an intracellular solution composing of (in mM) 130 KCl, 10 HEPES, 0.5 CaCl_2_, 10 EGTA, 2 MgCl_2_, 2 Mg-ATP and 0.3 Na-GTP at pH 7.3. CNQX (20 μM), tetrodotoxin (TTX 10 μM) and bicuculline (BIM 20 μM) were added into perfusion solution before recording to accurately verify the NMDAR miniature excitatory postsynaptic currents (mEPSC). In previous trials, AP-5 (a NMDA receptor antagonist, 2μM) was added after NMDA receptor- mEPSC was detected, and mEPSC fade away a few minutes later. Therefore, the mEPSC was mediated by NMDA receptors. ACSF without Mg^2+^ is used to observe NMDA receptor-mediated synaptic transmission. All responses were recorded by an EPC 10 amplifier and Pulse 8.52 software (HEKA, Germany). Current data was filtered with an eight-pole, low-pass Bessel filter at 2.9 kHz and digitized at 10 kHz for off-line analysis. mEPSC was then analyzed by Clampfit 9.0 (Axon Instruments, USA). Total charge transfer by all mEPSC was determined for each condition in 5 min and repeated 8 times.

### Statistical analysis

Statistical analysis was performed by SPSS 19.0 (IBM, Chicago, IL). All data were expressed as mean±standard deviation(SD). Time course data for both the thermal and mechanical hyperalgesia were analyzed by two-way ANOVA with repeated measures to detect interactions between treatments. ANOVAs with statistically significant interactions between treatments (P < 0.05) were followed by post hoc comparisons using Bonferroni’s *t* test when appropriate. The results of Western blot, immunohistochemistry, amplitude and interevent intervals of NMDAR-mediated mEPSC in different groups, one-way ANOVA followed by Tukey HSD test were performed. Statistical analysis was performed with GraphPad Prism 5.0 (GraphPad Software Inc, La Jolla, CA). A *P* value<0.05 was considered statistically significant.

## Results and discussion

### Remifentanil-induced postoperative mechanical and thermal hyperalgesia

There was no significant difference in the baseline PWT to mechanical stimuli and PWL to thermal stimuli among different groups (*P* >0.05). Compared with group C, PWT and PWL were significantly decreased at 2, 6, 24, 48 h in other groups except group D (*P*<0.01). Remifentanil infusion at the rate of 1.2 μg.kg^−1^.min^−1^ for 90 min or plantar incision significantly decreased PWT and PWL at 2, 6, 24 and 48 h after plantar incision (*P* < 0.01, S1 Fig). Compared with group I and group R, intraoperative administration of remifentanil during incision operation significantly decreased PWT and PWL (P < 0.01, S1 Fig). Dexmedetomidine administration individually had no effect on PWT and PWL compared with group C. However, dexmedetomidine administration before plantar incision induced a remarkable increase in PWT and PWL at 2, 6, 24, 48 h compared with group R+I (*P* <0.01, S1 Fig).

### Remifentanil-induced postoperative hyperalgesia increased the expression of NR1 and NR2B subunit in the plasma membrane, but not NR2A subunit in the spinal cord

Compared with group C, total (t) and membrane (m) expression of NR1, NR2B and the ratio of m/tNR1, m/tNR2B was significantly increased after incision or remifentanil infusion (*P* < 0.01, S2A, S2C and S2E Fig). The combination of surgery and remifentanil infusion could magnify the enhancement of the membrane and total expression of NR1 and NR2B subunits induced by incision or remifentanil respectively (P < 0.01, S2A, S2C and S2E Fig). Dexmedetomidine administration individually had no effect on NMDAR trafficking compared with group C. However, dexmedetomidine administration before plantar incision caused a significant reduction in the expression of mNR1, tNR1, mNR2B, tNR2B and the ratio of m/tNR1, m/tNR2B compared with group R+I (*P* <0.01, S2 Fig). No considerable changes were observed in mNR2A, tNR2A and the ratio of m/tNR2A (*P* > 0.05, S2C and S2D Fig).

The above data showed that remifentanil infusion and plantar incision might induce hyperalgesia by increasing membrane trafficking of NR1 and NR2B subunit, while dexmedetomidine might produce a preventive effect via inhibiting the trafficking process. The changes of NR1, NR2B expression detected by immunohistochemistry staining in dorsal horn were the same to that of Western blot (*P*< 0.05, S3B, S3C, S3D, S4B, S4C and S4D Figs), while the expression of NR2A was still the same (*P*>0.05, S3C and S4C Figs).

### Remifentanil-induced postoperative hyperalgesia increased the expression of PKCγ, CaMKIIα and pCaMKIIα in the spinal cord

Compared with group C, the results suggest that the expression of PKCγ, CaMKIIα and pCaMKIIα were significantly increased after remifentanil infusion or incision (*P* < 0.01, S5A, S5B, S5C, S5D and S5E Fig). Intraoperative administration of remifentanil during incision operation could magnify the enhancement of PKCγ, CaMKIIα and pCaMKIIα expression induced by incision or remifentanil respectively (P < 0.01, S5 Fig). Treatment with dexmedetomidine before plantar incision could significantly decrease the expression of PKCγ, CaMKIIα and pCaMKIIα compared with group R+I (*P* < 0.01, S5A, S5B, S5C, S5D and S5E Fig).

### Dexmedetomidine decreases remifentanil- induced NMDAR function enhancement in dorsal horn neurons

To further confirm the effect of dexmedetomidine on NMDAR -mediated mEPSC in spinal dorsal horn neurons, we examined NMDAR-mediated mEPSC with co-incubation of 4 nM remifentanil and 2 nM, 4 nM and 6 nM dexmedetomidine. Both frequency and amplitude of NMDAR- mediated mEPSC were dose-dependent decreased by dexmedetomidine compared with group R (*P* < 0.01, S6A, S6B and S6C Fig). The above data suggested that dexmedetomidine could attenuate NMDAR function enhanced by remifentanil in dorsal horn neurons.

The current study showed that remifentanil infusion and incision could induce thermal and mechanical hyperalgesia in a rat model of incisional pain, which was prevented by dexmedetomidine. The enhancement of membrane and total NR1 and NR2B subunits expression together with PKCγ, CaMKIIα and pCaMKIIα expression were observed in spinal dorsal horn after remifentanil infusion and incision, but there was no change in NR2A subunit. The expression was also decreased by dexmedetomidine. In whole-cell patch-clamp experiment, the amplitude and frequency of NMDAR- mediated mEPSC were enhanced by 4 nM remifentanil infusion but dose-dependent attenuated by dexmedetomidine. The above data suggested that α2AR agonist dexmedetomidine could depress remifentanil-induced hyperalgesia via regulating subunits trafficking and function of NMDAR as well as PKCγ, CaMKIIα and pCaMKIIα expression in spinal dorsal horn.

The rat model of incisional pain was performed in accordance with Brennan described to simulate remifentanil-induced postoperative hyperalgesia [18]. The reason why sevoflurane was chosen as the anesthetic through the entire experiment was because it had no impact on behavioral tests [9]. The dosage of remifentanil was a key factor in present study. Remifentanil infusion at a rate of 1.2 μg.kg^−1^.min^−1^ could evoke hyperalgesia in rats, and continuous infusion for 60~90 min led to significantly reduction of mechanical pain threshold [9, 10, 19]. Therefore, remifentanil was infused at a rate of 1.2 μg.kg^−1^.min^−1^ for 90 min in this study. It was tried out that PWT of tissues around the incision was prolonged than that of incisional group after 50 μg/kg dexmedetomidine was injected subcutaneously. Several studies demonstrated that hyperalgesia induced by remifentanil occurred at 2 h and reached the peak at 24–48 h after administration [10, 20]. Combination of remifentanil infusion and incision could cause greater PWT and PWL compared with remifentanil infusion or incision alone in our previous studies [9, 10, 19]. Our behavioral results suggested that either remifentanil infusion or surgery alone induced hyperalgesia to mechanical and thermal stimuli, which was greater when remifentanil administration and surgery are combined. To conduct a better research about the role of dexmedetomidine, we set remifentanil infusion and incision group as the pain group. We did the experiment about dexmedetomidine and results suggested that dexmedetomidine did not change PWT and PWL at the dose of 50 μg/kg. It suggested that the dose of dexmedetomidine had no effect on pain process.

The whole cell patch clamp was used to detect NMDAR-mediated mEPSC of neurons in spinal dorsal horn to distinguish presynaptic and postsynaptic response. On the basis of synaptic vesicular release quantum theory, miniature postsynaptic currents are supposed to represent the spontaneous release of neurotransmitter or vesicles from presynaptic membrane. The frequency of NMDAR-induced mEPSC is considered as the presynaptic effects, whereas the amplitude is thought to reflect postsynaptic effects [21, 22]. Therefore, the function of NMDAR was evaluated by the frequency and amplitude of NMDAR-mediated mEPSC [21]. 4 nM remifentanil for 60 min increased both frequency and amplitude of NMDAR-mediated mEPSC significantly in previous study. We did the experiment about dexmedetomidine and results suggested that dexmedetomidine alone did not change NMDAR-mediated mEPSC. However, both frequency and amplitude of NMDAR- mediated mEPSC enhanced by remifentanil were dose-dependent decreased by dexmedetomidine. The above data suggested that dexmedetomidine could attenuate NMDAR function enhanced by remifentanil in dorsal horn neurons. The results that up-regulation of NR1 and NR2B subunits membrane trafficking was consistent with function of mEPSCs recorded.

Numerous studies were conducted to explore the mechanism of OIH, but there were still different viewpoints on it. The majority of academics suggest that it is associated with acute receptor desensitization caused by G-protein receptor decoupling, upregulation of cAMP pathway and NMDAR activation [23]. NMDAR activation accounts for OIH and tolerance processes, which is in accord with that NMDAR antagonist ketamine was able to inhibit central sensitization stated in experimental studies performed in both animals and volunteers [24]. So NMDAR has recently been considered to play a crucial role in synaptic plasticity and chronic pain formation [24]. The NR2 subunits amplify NR1 activity and these subunits co-assemble in various combinations to induce functional variability in NMDAR signaling [25]. The up-regulation of expression and membrane trafficking of NR1 and NR2B subunits in spinal cord after remifentanil administration and incision verified in the experiment was in consistent with our previous results [9, 10]. The results showed that remifentanil infusion and plantar incision might induce hyperalgesia by increasing the expression and membrane trafficking of NR1 and NR2B subunit detected by western blot and immunohistochemistry staining. It is worth noting that NR2B subunit plays a dominant role in sensory pathway of spinal dorsal horn. Moreover, there is a substantial amount of evidence that tyrosine phosphorylation of NR2B on distal C-terminus at Tyr1472 in spinal dorsal horn may contribute to NMDAR activation and development of OIH [20, 26].

It has been proposed that Ca^2+^ influx via activation of NMDAR results in PKCγ and CaMKIIα activation as well as CaMKIIα autophosphorylation at position Thr286, leading to NMDAR phosphorylation and activation and thus Ca^2+^ influx through the channels [8, 25, 27, 28]. The positive feedback would potentiate PKCγ and CaMKIIα activity and intracellular Ca^2+^ level, leading to neuronal excitability [29]. Furthermore, PKCγ indirectly potentiates NMDAR hypersensitization by activating a downstream signalling peptide of the tyrosine kinase (Src) signalling cascade [27, 30, 31]. Thus, translocation of PKC from cytoplasm to cell membrane is a sensitive indicator of activation [32]. Increasing evidence indicates that the distribution and function of NMDAR is modulated by PKC via regluating the interactions between NMDAR, postsynaptic density (PSD) and cytoskeletal proteins [33]. PKC activation increases substance P as well [34]. S1303 and S1323 serine residues in C-terminal region of the NR2B subunit are directly phosphorylated by PKC and enhance the PKC-mediated NR2B/NR1 currents [27, 30]. In addition, S1303 had been confirmed as a major site of CaMKII phosphorylation [27]. Neurogranin (Ng), previously known as RC3 or P17, is a postsynaptic neuron specific protein that serve as a link between PKC and CaMKII [35]. A large increase in intracellular Ca^2+^ prompts the release of CaM by dissociating CaM/Ng complex. Latter in the presence of Ca^2+^, Ca^2+^/CaM-dependent enzymes such as CaMKII is activated [36]. In contrast, low Ca^2+^ level favors CaM/Ng formation and activates CaMKII [37]. Alternatively, CaM/Ng complex may also be dissociated via Ng phosphorylation by PKC [36, 38]. OIH was absent in mice lacking gene coding for PKCγ and CaMKIIα T286A mutant mice [39]. Pretreatment with the PKCγ agonist resulted in CaM release, pCaMKII formation and development for OIH.

The α2 adrenergic receptor agonist dexmedetomidine may act on spinal α2 receptor. α2 adrenoceptor is also G-protein coupled receptors. They exert their effects through inhibition of cAMP formation and ultimately reduction in PKA activity once activated. Spinal PKA activation enhanced the activity of Fyn kinase that is major tyrosine kinase phosphorylates NR2B at Tyr1472, and then potentiate NMDARs functions [26, 40]. Anti-hyperalgesic effect of dexmedetomidine may depend on its ability to modulate spinal NMDAR activation by suppression of NR2B phosphorylation via a Fyn-dependent mechanism. It was in consistent with our previous results that dexmedetomidine might produce a preventive effect via inhibiting the NR1 and NR2B subunits trafficking process. And thus reduction in Ca^2+^ influx resulted in decreased PKC activation, CaMKIIα activation and autophosphorylation. In addition, several studies demonstrated that dexmedetomidine had a high affinity for I_2_ imidazole receptors. Both α2 adrenergic receptor and I_2_ imidazole receptors could provide analgesic effects in spinal cord.

## Conclusions

In conclusion, the present study indicates that antihyperalgesic effects of dexmedetomidine may be associated with enhanced membrane trafficking and expression of NMDAR, expression of PKC and CaMKII and current in both presynaptic and postsynaptic levels. Dexmedetomidine may be a potential new drug target to treat remifentanil-induced hyperalgesia.

## Supporting information

S1 Fig Effects of dexmedetomidine on remifentanil-induced mechanical and thermal hyperalgesia. Remifentanil was infused intravenously at the rate of 1.2 μg.kg^−1^.min^−1^ for 90 min in group R, group R+I and group D+R+I. Dexmedetomidine was injected subcutaneously at the dose of 50μg/kg in group D and group D+R+I. PWL (A) and PWT (B) were evaluated at −24 h, 2 h, 6 h, 24 h and 48 h after infusion. Data were analyzed by repeated measures ANOVA and expressed as mean±SD. Compared with the group C, *P < 0.01; compared with the group R, ^#^P < 0.01; compared with the group I, ^$^P < 0.01; compared with the group R+I, ^&^P < 0.01; compared with the group D+R+I, ^§^P < 0.01; N = 8, analysis of variance.
(TIF)

S2 Fig Effects of dexmedetomidine on expression of NR1, NR2A and NR2B subunits by western blot. Membrane trafficking of NR1 and NR2B subunits was increased after remifentanil administration and incision, while dexmedetomidine might produce a preventive effect. The spinal cord L4–L5 segments were removed after the last behavioral test for western blot. EGFR and β-actin were internal control. The band intensity of group C was assigned a value of 1. Bands of membrane and total NR1, NR2A, NR2B protein were detected by Western blot (A, C, E). (B) Bar chart of the ratios of mNR1/EGFR, tNR1/β-actin and m/tNR1. (D) The ratios of mNR2A/EGFR, tNR2A/β-actin and m/tNR2A. (F) The ratios of mNR2B/EGFR, tNR2B/β-actin and m/tNR2B. Data were analyzed by ANOVA and expressed as mean±SD. Compared with the group C, *P < 0.01; compared with the group R, ^#^P < 0.01; compared with the group I, ^$^P < 0.01; compared with the group R+I, ^&^P < 0.01; compared with the group D+R+I, ^§^P < 0.01; N = 8, analysis of variance.
(TIF)

S3 Fig Effects of dexmedetomidine on expression of NR1, NR2A and NR2B subunits by immunohistochemistry staining. The L4-L5 segments of spinal cords were collected after the last behavioral testing for immunohistochemistry. When compared with group C, NR1 and NR2B expression is dramatically increased after remifentanil infusion with incision; while the process was significantly suppressed by pretreatment with dexmedetomidine(A). No considerable changes were observed in NR2A subunit (A). Representative photomicrographs of the L4-6 spinal cord are shown here (Scale bar = 50μm).
(TIF)

S4 Fig Effects of dexmedetomidine on expression of NR1, NR2A and NR2B subunits by immunohistochemistry staining. Mean IOD of NR1, NR2A and NR2B subunits were calculated by IPP software (S4 Fig B, C, D). Data were expressed as mean±SD. Compared with the group C, *P < 0.01; compared with the group R, ^#^P < 0.01; compared with the group I, ^$^P < 0.01; compared with the group R+I, ^&^P < 0.01; compared with the group D+R+I, ^§^P < 0.01; N = 8, analysis of variance.
(TIF)

S5 Fig Effects of dexmedetomidine on expression of PKCγ, CaMKIIα and pCaMKIIα by western blot. The expression of PKCγ, CaMKIIα and pCaMKIIα were significantly increased after remifentanil infusion and incision, while the phenomenon was prevented by dexmedetomidine. The L4–L5 segments of spinal cords were removed after the last behavioral test for western blot. β-actin was internal control. The band intensity of group C was assigned a value of 1. Bands of PKCγ, CaMKIIα and pCaMKIIα protein by Western blot(A, C). (B) Bar chart of the ratios of PKCγ/β-actin. (D) Bar chart of the ratios of CaMKIIα/β-actin. (E) Bar chart of the ratios of pCaMKIIα/β-actin. Data were analyzed by ANOVA and expressed as mean±SD. Compared with the group C, *P < 0.01; compared with the group R, ^#^P < 0.01; compared with the group I, ^$^P < 0.01; compared with the group R+I, ^&^P < 0.01; compared with the group D+R+I, ^§^P < 0.01; N = 8, analysis of variance.
(TIF)

S6 Fig Dexmedetomine dose-dependent prevents NMDAR-mediated mEPSCs enhanced by remifentanil in dorsal horn neurons. In the presence of TTX (10 μM), GABAR antagonist bicuculline (BIM, 20 μM) and AMPAR antagonist CNQX (20 μM), NMDAR-mediated mEPSCs were recorded at the holding potential of −70 mV. Representative traces of mEPSCs under blank control (group C), remifentainil 4 nM(group R), dexmedetomine 4 nM(group D), dexmedetomine 2 nM + remifentainil 4 nM(group D1), dexmedetomine 4nM + remifentainil 4 nM(group D2), dexmedetomine 6 nM + remifentainil 4 nM(group D3) showed in graph A. Scale bar, 100 pA, 30 s. The Bar chart of mEPSCs frequency and mEPSCs amplitude were showed in graph B and C. Data were expressed as mean±SD. Compared with the C group, *P < 0.01; compared with the R group, ^#^P < 0.01; compared with the D1 group, ^$^P < 0.01; compared with the D2 group, ^&^P < 0.01; compared with the D3 group, ^§^P < 0.01; N = 8, analysis of variance.
(TIF)
