# Supplementary figures and images for: Prevention of Remifentanil Induced Postoperative Hyperalgesia by Dexmedetomidine via Regulating the Trafficking and Function of Spinal NMDA Receptors as well as PKC and CaMKII Level In Vivo and In Vitro
Source: PLoS One. 2017 Feb 9;12(2):e0171348. doi: 10.1371/journal.pone.0171348 (PMC5300256; doi:10.1371/journal.pone.0171348)

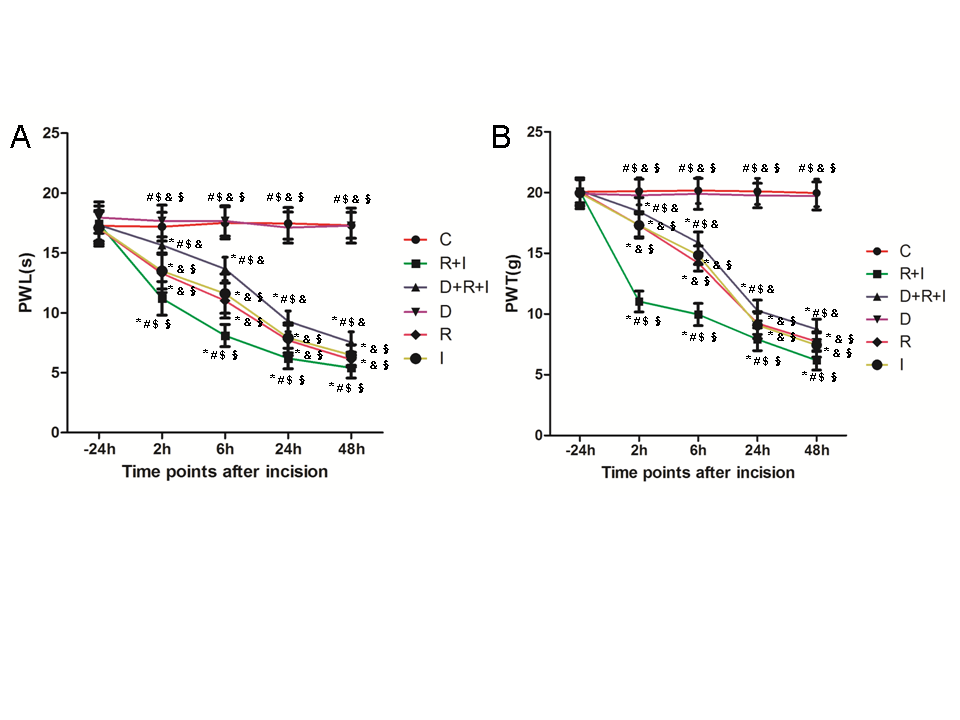

Supplement: S1 Fig — Remifentanil was infused intravenously at the rate of 1.2 μg.kg−1.min−1 for 90 min in group R, group R+I and group D+R+I. Dexmedetomidine was injected subcutaneously at the dose of 50μg/kg in group D and group D+R+I. PWL (A) and PWT (B) were evaluated at −24 h, 2 h, 6 h, 24 h and 48 h after infusion. Data were analyzed by repeated measures ANOVA and expressed as mean±SD. Compared with the group C, *P < 0.01; compared with the group R, #P < 0.01; compared with the group I, $P < 0.01; compared with the group R+I, &P < 0.01; compared with the group D+R+I, §P < 0.01; N = 8, analysis of variance. (TIF) [file pone.0171348.s001.tif]

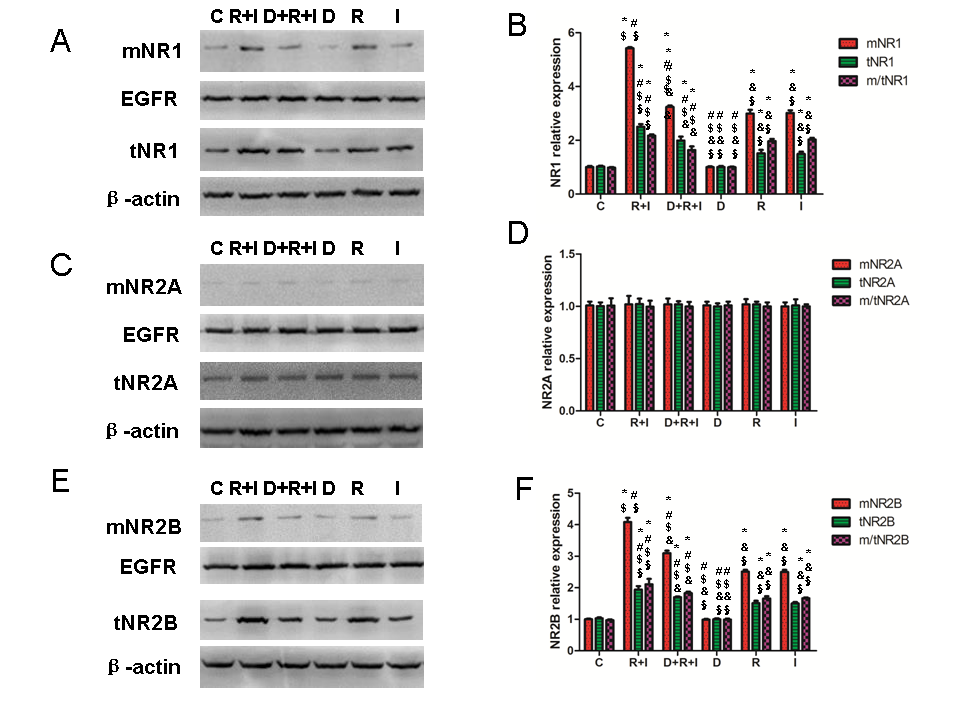

Supplement: S2 Fig — Membrane trafficking of NR1 and NR2B subunits was increased after remifentanil administration and incision, while dexmedetomidine might produce a preventive effect. The spinal cord L4–L5 segments were removed after the last behavioral test for western blot. EGFR and β-actin were internal control. The band intensity of group C was assigned a value of 1. Bands of membrane and total NR1, NR2A, NR2B protein were detected by Western blot (A, C, E). (B) Bar chart of the ratios of mNR1/EGFR, tNR1/β-actin and m/tNR1. (D) The ratios of mNR2A/EGFR, tNR2A/β-actin and m/tNR2A. (F) The ratios of mNR2B/EGFR, tNR2B/β-actin and m/tNR2B. Data were analyzed by ANOVA and expressed as mean±SD. Compared with the group C, *P < 0.01; compared with the group R, #P < 0.01; compared with the group I, $P < 0.01; compared with the group R+I, &P < 0.01; compared with the group D+R+I, §P < 0.01; N = 8, analysis of variance. (TIF) [file pone.0171348.s002.tif]

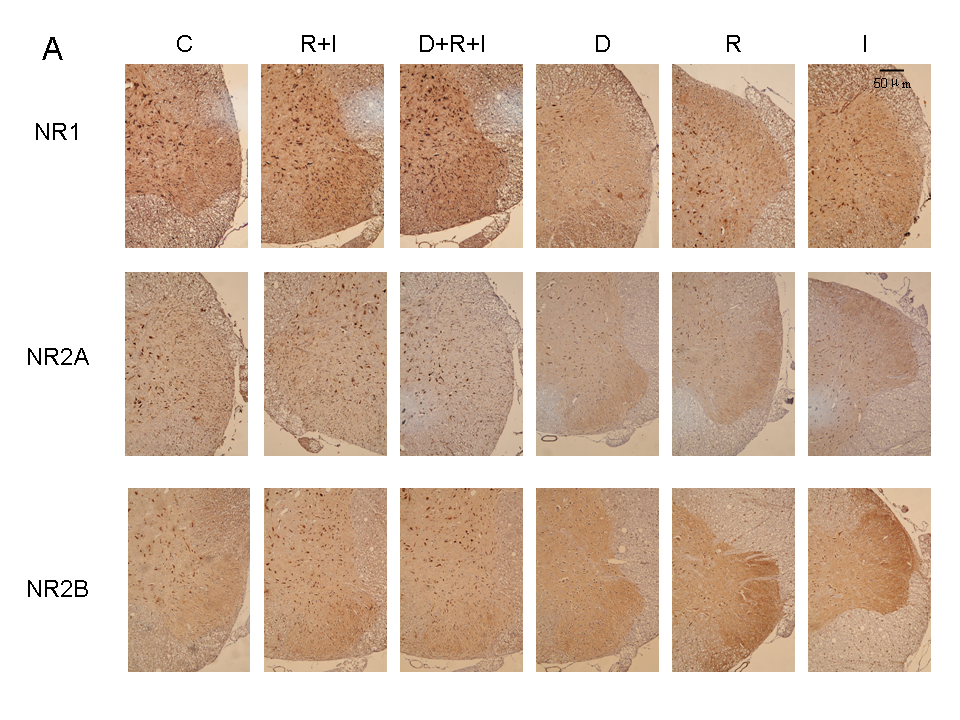

Supplement: S3 Fig — The L4-L5 segments of spinal cords were collected after the last behavioral testing for immunohistochemistry. When compared with group C, NR1 and NR2B expression is dramatically increased after remifentanil infusion with incision; while the process was significantly suppressed by pretreatment with dexmedetomidine(A). No considerable changes were observed in NR2A subunit (A). Representative photomicrographs of the L4-6 spinal cord are shown here (Scale bar = 50μm). (TIF) [file pone.0171348.s003.tif]

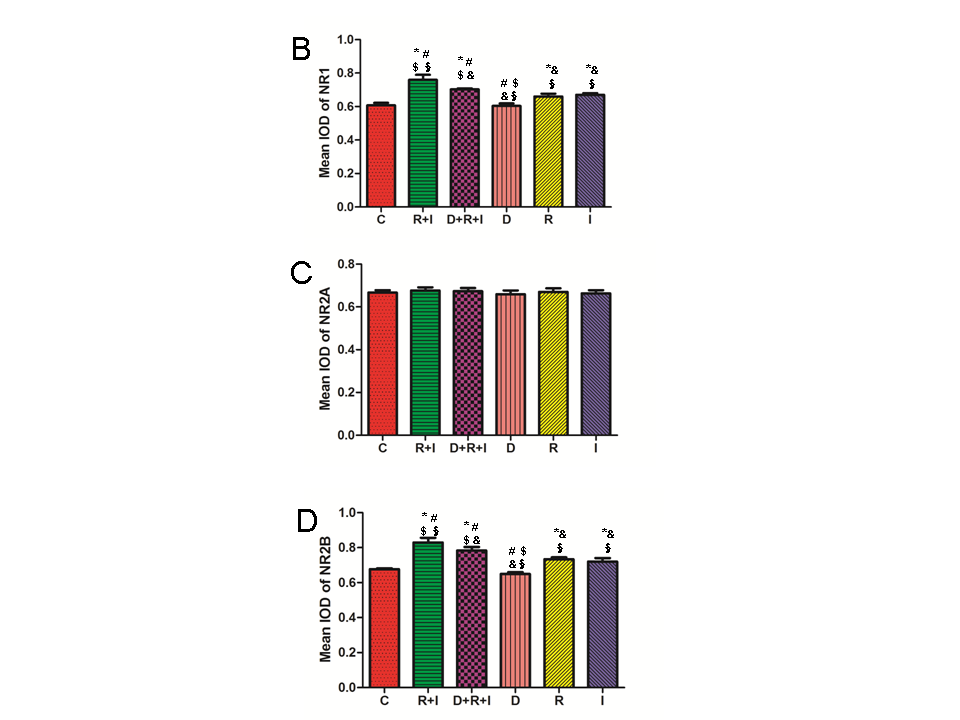

Supplement: S4 Fig — Mean IOD of NR1, NR2A and NR2B subunits were calculated by IPP software (S4 Fig B, C, D). Data were expressed as mean±SD. Compared with the group C, *P < 0.01; compared with the group R, #P < 0.01; compared with the group I, $P < 0.01; compared with the group R+I, &P < 0.01; compared with the group D+R+I, §P < 0.01; N = 8, analysis of variance. (TIF) [file pone.0171348.s004.tif]

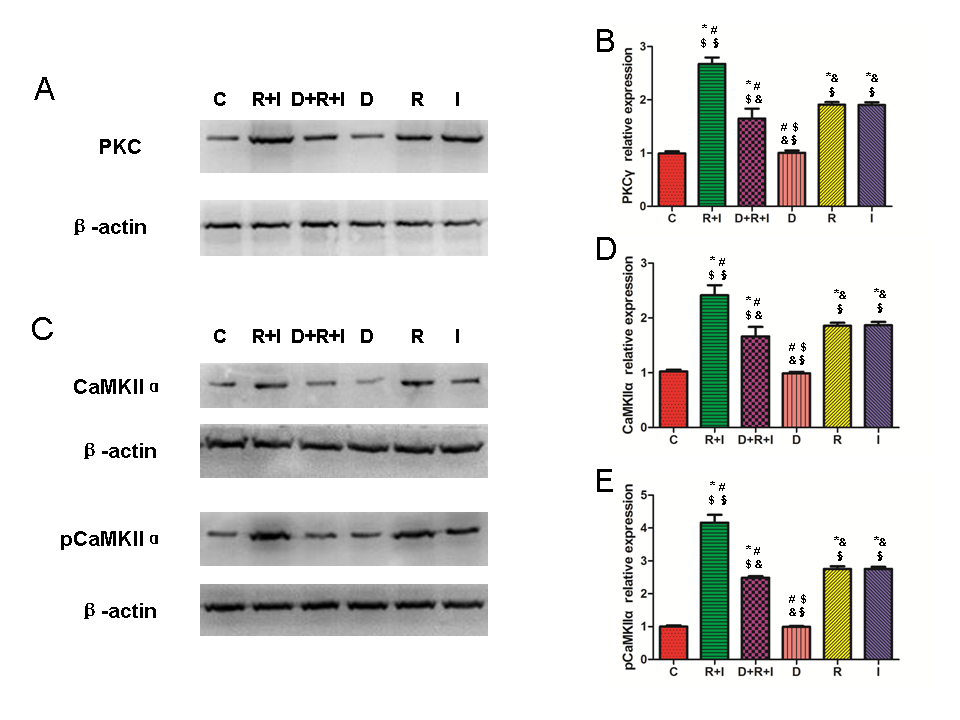

Supplement: S5 Fig — The expression of PKCγ, CaMKIIα and pCaMKIIα were significantly increased after remifentanil infusion and incision, while the phenomenon was prevented by dexmedetomidine. The L4–L5 segments of spinal cords were removed after the last behavioral test for western blot. β-actin was internal control. The band intensity of group C was assigned a value of 1. Bands of PKCγ, CaMKIIα and pCaMKIIα protein by Western blot(A, C). (B) Bar chart of the ratios of PKCγ/β-actin. (D) Bar chart of the ratios of CaMKIIα/β-actin. (E) Bar chart of the ratios of pCaMKIIα/β-actin. Data were analyzed by ANOVA and expressed as mean±SD. Compared with the group C, *P < 0.01; compared with the group R, #P < 0.01; compared with the group I, $P < 0.01; compared with the group R+I, &P < 0.01; compared with the group D+R+I, §P < 0.01; N = 8, analysis of variance. (TIF) [file pone.0171348.s005.tif]

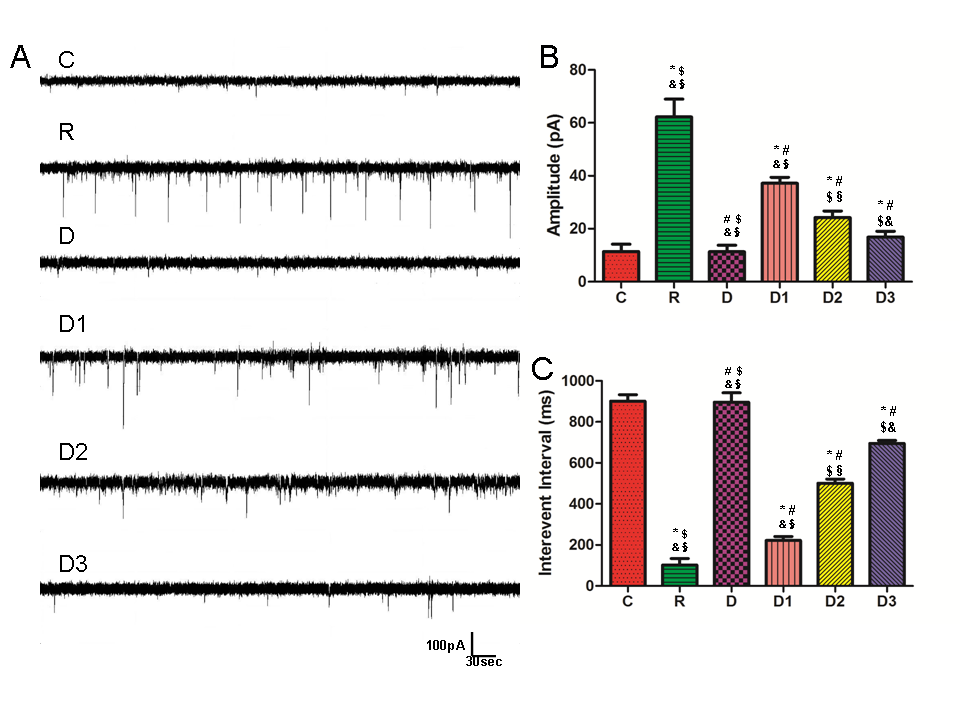

Supplement: S6 Fig — In the presence of TTX (10 μM), GABAR antagonist bicuculline (BIM, 20 μM) and AMPAR antagonist CNQX (20 μM), NMDAR-mediated mEPSCs were recorded at the holding potential of −70 mV. Representative traces of mEPSCs under blank control (group C), remifentainil 4 nM(group R), dexmedetomine 4 nM(group D), dexmedetomine 2 nM + remifentainil 4 nM(group D1), dexmedetomine 4nM + remifentainil 4 nM(group D2), dexmedetomine 6 nM + remifentainil 4 nM(group D3) showed in graph A. Scale bar, 100 pA, 30 s. The Bar chart of mEPSCs frequency and mEPSCs amplitude were showed in graph B and C. Data were expressed as mean±SD. Compared with the C group, *P < 0.01; compared with the R group, #P < 0.01; compared with the D1 group, $P < 0.01; compared with the D2 group, &P < 0.01; compared with the D3 group, §P < 0.01; N = 8, analysis of variance. (TIF) [file pone.0171348.s006.tif]
